# Salivary Vasopressin as a Potential Non–Invasive Biomarker of Anxiety in Dogs Diagnosed with Separation–Related Problems

**DOI:** 10.3390/ani9121033

**Published:** 2019-11-26

**Authors:** Federica Pirrone, Ludovica Pierantoni, Andrea Bossetti, Stefania Uccheddu, Mariangela Albertini

**Affiliations:** 1 Department of Veterinary Medicine, University of Milan, via Celoria 10, 20133 Milan, Italy; andrea.bossetti@gmail.com (A.B.); mariangela.albertini@unimi.it (M.A.); 2 Veterinary Behaviour & Consulting Services at CAN Training Centre, 80128 Naples, Italy; ludovica.pierantoni@gmail.com; 3 Vet Ethology, 3090 Overijse, Belgium; uccheddus@gmail.com

**Keywords:** dogs, separation distress disorder, vasopressin, oxytocin, anxiety

## Abstract

**Simple Summary:**

Oxytocin and vasopressin have been shown to have opposite effects on the expression of anxiety and fear responses in rodents. In the present study, we analyzed the salivary fluctuations of these neuropeptides in both behaviorally normal dogs and dogs with separation distress in response to a three-minute separation from the owner, in a new environment. Dogs with a previous diagnosis of separation distress showed more anxiety-related behaviors and higher concentrations of vasopressin than control dogs when separated from the owner. Further research is needed on the potential use of salivary vasopressin as an early, non-invasive biomarker of anxiety-related disorders in pet dogs.

**Abstract:**

Physiological biomarkers of canine anxiety have not been extensively investigated to date. To identify new biomarkers in dogs, we compared behaviorally normal dogs (Control group, N = 13) to dogs diagnosed with separation problems (Case group, N = 13) as they were introduced into a novel environment in the presence of two strangers and subjected to a short episode of separation and reunion with the owner. During the separation phase, dogs in the Case group explored significantly less than controls and were significantly more persistent in expressing passive stress-coping strategies aimed at seeking proximity to their owners. When the owners returned, dogs with separation distress spent significantly more time jumping up on the strangers than control dogs did. Salivary oxytocin and vasopressin concentrations did not differ between samples taken before and after the separation. However, vasopressin concentrations immediately after separation were significantly higher in the Case than in the Control group and remained higher, although not significantly so, 10 min later. These results indicated that dogs with separation distress became more anxious than typical dogs when separated from their owner in an unfamiliar environment and provided preliminary support for the use of salivary vasopressin as a possible biomarker for anxiety-related responses in dogs.

## 1. Introduction

Both humans and dogs are highly social mammals who develop selective forms of sociality, in which are embedded lasting relationships defined as social bonds or attachments [1]. An attachment bond may also link dogs and owners [2], the latter becoming the animals’ reference point in the environment, influencing their welfare [3]. The attachment relationship between dogs and owners is one of the primary reasons why people keep dogs as companion animals [4].

Studies throughout the last decade have explored dogs’ attachment-related behavioral reactions to separation from and reunion with the owner [2,3,5], while little is known about concomitant physiological reactions. Physiological changes, which include increased secretion of glucocorticoids and/or adrenocorticotropic hormone (ACTH), as well as altered cardiovascular or immune parameters, have been described in dogs during laboratory testing [4,6]. Increased circulating levels of nerve growth factor (NGF) have been directly associated with psychosocial stress in human and animal models [7,8]. However, in both rodents and humans, the closely related neuropeptides oxytocin (OT) and vasopressin (AVP) have been associated with distinct, partly opposite roles in social behavior, as well as in stress, fear, and anxiety responsiveness following social separation [9,10,11]. In particular, OT has been found to attenuate anxiety, central fear responses, and neuroendocrine reactivity [12], while stimulation of the AVP system has been shown to lead to augmented anxiety and fear expression [10] and increased neuroendocrine stress response [12].

Recently, some studies have begun to focus on OT in affiliative interactions in dog-human dyads [13,14]. We are also aware of two studies investigating the relationships between AVP and behavior in dogs, both of which revealed positive associations with fear and aggression [15,16].

To the best of our knowledge, there are no published data on the roles of these neuropeptides in anxiety-like behavior in dogs.

This study sought to examine the behavior and OT and AVP fluctuations in dogs in response to a commonly occurring human-dog interaction that might potentially induce anxiety in dogs. Specifically, we compared two groups of pet dogs that were separated from their owners for 3 min and then reunited with them, all while they were in a novel environment and the presence of two unfamiliar people. The Control group consisted of behaviorally normal dogs, while dogs diagnosed with separation-related problems (SRP) formed the Case group. Separation-related problems are described as physical, physiological, and/or behavioral signs of the distress exhibited by the dog only in the absence of, or lack of access to, the owner [17].

Approximately 14%–20% of dog patients [18,19] from general veterinary practices show signs of SRP in their owners’ absence [20], and the anxiety emotional system is recognized as one possible cause. However, as with other anxiety disorders in veterinary patients, underdiagnosis of anxiety-dependent separation problems has been previously reported [21] due to misinterpretation of normal and pathological anxiety [22] and the lack of recognition of mild clinical signs by owners [23]. Therefore, unsurprisingly, according to Karagiannis et al. [24], it is suspected that up to 50%–56% of the overall dog population may actually display clinical symptoms of SRP at some point in their life [25,26], which, out of the total population of approximately 160 million dogs in the US and Europe [27,28] represents approximately 85 million dogs with SRP on the two continents. Underdiagnosis of SRP suggests a need to identify putative measurable markers that are specific to these behaviors, which would allow early diagnosis and intervention. Since separation-related problems are one of the primary cited reasons for the relinquishment of dogs to animal shelters [29,30], the relevant practical implications make research in this direction unquestionably worthwhile.

To assess the levels of relevant hormones, we examined their concentrations in saliva. A recent study by MacLean [31] validated salivary measures of OT and AVP in dogs. Because saliva collection is simple and painless, it is better suited for assessments in dogs than other methods, such as using plasma or urine, which present a host of challenges related to design, analysis, and welfare [31]. However, it is currently unknown whether these measures respond dynamically to anxiety-related aspects of human-dog interaction.

From previous findings [4], we hypothesized that separation from the owner in our experimental setting would not lead to different behavioral responses from dogs in the two groups. In fact, using an experimental protocol similar to ours, Parthasarathy and Crowell–Davis [4] found that, regardless of their separation anxiety status, all of the dogs displayed behavioral signs of increased anxiety when their owners left the room. Conversely, from observations in rodent and human models, we expected differences in endocrine responses during the separation phase, with a lower level of OT and a higher concentration of AVP in the Case group than in the Control group.

## 2. Materials and Methods 

The Animal Welfare Committee (OPBA) of the University of Milan approved this research (OPBA_106_2018). All methods were performed in accordance with the current European and Italian legislation.

### 2.1. Participants and Study Setting

This study was part of a research project designed to discover novel potential biomarkers of stress and anxiety in pet dogs brought by their owner to the Veterinary Behavior and Consulting Services at CAN (Comportamento Animale Napoli) Training Center in Naples, Italy, for signs of fear or anxiety-related conditions. Initially, the owners of all dogs were asked, through a brief telephone interview, the reason for requesting the behavioral visit. Dogs were then tested, and, for this study, 13 dogs were selected by simple random sampling from those who had SRP according to the owners and received a diagnosis that confirmed SRP based on the outcome of the behavioral visit (Case group). 

We also selected 13 controls from dogs whose owners attended the training center for the first time to get information on the service of dog’s night board and daycare. The target sample size for the study was determined on the basis of a power analysis with 80% power, a large effect size (0.50), and an α value (error rate) of 0.05. The selected controls, who were rated as behaviorally typical based on the outcome of the visit purposely performed to include them in the present study, were similar to the cases with respect to the owners’ gender and the dogs’ sex, age, origin, and breed type (Supplementary Table S1). The dogs were 12 females (of which 4 were spayed), and 14 males (of which 6 were neutered), either pure or mixed breed. The mean age of the dogs, in years, was 3.7 (SE = 0.4, range: 1–8). Moreover, cases and controls had belonged to their current owners for at least one year to ensure that they had a reasonable amount of time to form a relationship with the owners and were an adult at the time of the study (1–10 years). All dogs underwent a physical examination. The Case group also had blood tests done. Those who were healthy for the veterinarian and not in treatment for behavioral or physical problems at the time of data collection were included in the study. Exclusion criteria included estrus, pregnancy, and nursing, as well as a history or present signs of stranger-directed aggression or fear of strangers or novel environments. 

**Figure 1:**
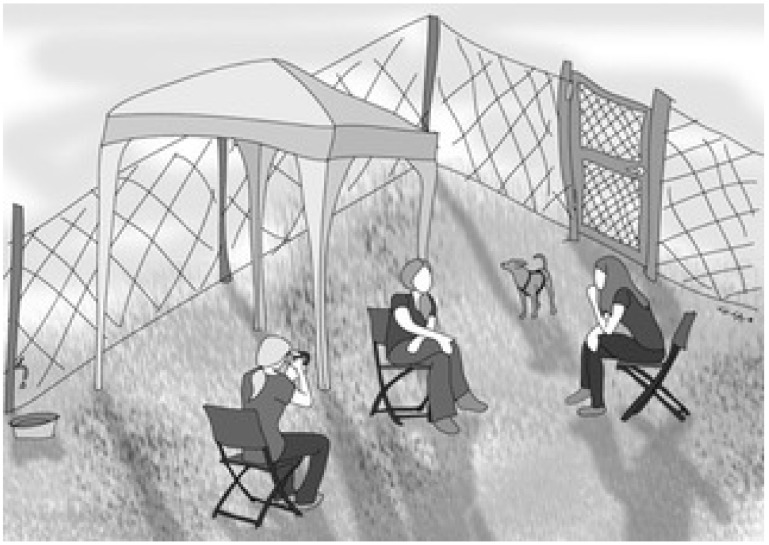
The spatial arrangement of the test. A moment during the session with one of the dogs. The owner and the two strangers (the veterinary behaviorist and the camerawoman) are visible. Drawing by Valentina Sammartano.

The experimenter explained the general objective of the research to all owners, who signed written informed consent and provided their assent to participate in the study. By agreeing to participate, owners confirmed that they were informed of a cameraperson’s presence for the videotaping procedure and acknowledged that, in compliance with the General Data Protection Regulation (GDPR) (EU) 2016/679, their data and video recordings would be stored on the principal investigator’s computer at the Department of Veterinary Medicine at the University of Milan and otherwise processed by the investigators of this study, with all ensuring an adequate level of data protection. Informed consent was obtained from the owners of dogs whose information, images, and/or videos would be published in an online journal, which could lead to their identification. Testing took place during the behavioral visit in a 300 m^2^ outdoor arena (Figure 1), containing three chairs (for the owner, the veterinary behaviorist, and a cameraperson), a bowl for fresh water, and some toys. The arena had four sides and was enclosed with chain-link fencing approximately 2 m tall; one side of the arena included an entrance gate. The duties of the veterinary behaviorist and the cameraperson were always performed by the same two women, who had never met the dogs before. The arena was located in a larger area. For standardization, and to minimize distractions and background noises, which could have acted as confounders, all dogs were tested in mild weather conditions and the late afternoon (between 1630 and 1830), after the center’s planned closure, so no other social stimuli were present.

### 2.2. Study Protocol 

Owners were instructed not to provide their dogs with food or exercise 1.5 hours prior to the start of the test. A simplified version of the Ainsworth’s strange situation test (ASST) was employed. During the test, the owner and veterinary behaviorist could talk to each other and interact with the dog only if he/she was seeking their attention. The cameraperson was instructed not to interact with the dog or with the veterinary behaviorist and owners to avoid reinforcing attention-seeking behaviors and to control for the possibility that the strangers would act differently around different dogs [4]. The owners were told that if at any point they were concerned about their dogs’ welfare or safety, they could stop the test. The entire procedure comprised three steps: An acclimatization phase, a separation phase, and a reunion phase. 

*Acclimatization phase (10 min).* Prior to the short separation task, the owner, the dog, the veterinary behaviorist, and the camerawoman entered the arena. The dog was left unleashed and free to explore the environment, while the owner and the strangers remained seated in the chairs. Saliva was collected from the dog at the end of the tenth minute (T0).

*Separation phase (3 min).* Immediately afterward, the owner left the arena, where the dog remained in the company of the two strangers. During the ASST, the veterinary behaviorist attempted to engage the dog in friendly interaction, including gently petting him/her and speaking to him/her in a calm tone (Supplementary Video S1). However, the veterinary behaviorist allowed the dog to lead these interactions, and dogs were always free to disengage and move away from her. If a dog exhibited signs of severe distress or anxiety, the owner was asked to come back, and the testing stopped (Supplementary Video S2). At the end of the third minute, the owner returned to the arena, sat in the same chair as before, and made conversation with the veterinary behaviorist, and the second sample of saliva was collected (T1). 

*Reunion phase (10 min).* Immediately upon returning, the owner was allowed to respond to his/her dog’s greeting by interacting both verbally and physically in a calm way. After ten minutes, saliva was collected (T2), and the test ended.

### 2.3. Parameters Recorded: Behavioral Responses

Observer-blind analysis of behavior was carried out with focal animal sampling and continuous recording using the Observer XT software package (Noldus Information Technology, 6702 EA Wageningen, The Netherlands). Another coder, expert in animal behavior but unfamiliar with the aims and conditions of the study, verified the reliability of the coding in 20% of the videos. A Cronbach’s α of 0.80 or higher was considered acceptable for this study. According to a study by Mariti et al. [2], the behaviors were divided into social and non–social, and each social behavior towards the owner and the strangers was analyzed (Table 1). Behavior definitions were formulated on the basis of a literature review [2,3,4,32,33,34,35,36,37]. As for social behaviors, attention-seeking and proximity were grouped to create the category spontaneous interactions. During the separation phase, we also recorded the interactions solicited by the veterinary behaviorist from the dogs, which included talking to and petting the dogs to comfort them if they showed signs of distress. Behavioral variables were measured in terms of relative frequency (the number of occurrences per minute) and/or duration (time spent on a behavior, expressed in seconds) of occurrence during each observation period.

### 2.4. Parameters Recorded: Endocrine Responses

We collected saliva samples from dogs using commercially available swabs (SalivaBio Children’s Swab, Salimetrics, Carlsbad, CA, USA). All samples were taken by the veterinary behaviorist. The swab was gently placed into the cheek or under the tongue of the dog for approximately 60 seconds, without the restraint of the animal. The dog’s salivation was stimulated by presenting the odor of food treats. The dog received a food treat only after the last saliva sample was taken because the consumption of food immediately before sample collection has been found to affect OT and AVP measurements [31]. Each sample was replaced in the device tube and closed with a plastic stopper to prevent evaporation. The collected material was refrigerated at 4 °C and then stored at −20 °C immediately after it arrived at the laboratory. At the time of analysis, the samples were thawed at room temperature and centrifuged according to the protocol for salivary samples. The laboratory technician who performed these analyses was blinded to the hypotheses and conditions. All samples were analyzed by enzyme-linked immunosorbent assay (ELISA) following previously validated protocols [31,34]. For the measurement of OT and AVP, we used commercially available enzyme-linked immunosorbent assay kits from Arbor Assays (Ann Arbor, MI, USA) and MyBiosource Inc. (San Diego, CA, USA). Each sample was prepared in duplicate, and concentrations were calculated using a Labisystem Multiskan Ex (Nepean, ON, Canada) microplate reader according to the relevant standard curves.

The mean recovery was 102.8% ± 10.8 for OT and 94.3 ± 2.2% for AVP. The average intra- and inter-assay coefficients of variation, respectively, were 4.7% and 8.8% for OT and 5.7% and 6.5% for AVP. The assay sensitivity was 17 pg/ml and 1 pg/ml for OT and AVP, respectively.

The laboratory technician was blinded to the hypotheses and conditions.

### 2.5. Statistical Analysis

Due to the number of animals and the distribution of the data, non-parametric statistics were used to analyze the behavioral and hormonal data [38,39]. Differences between groups were analyzed using the Mann–Whitney *U* test, while the Kruskal–Wallis test for multiple comparisons was conducted to compare behaviors within each group. A post hoc Mann–Whitney *U* test with the Bonferroni correction followed the Kruskal–Wallis test in case a significant effect was detected. The Friedman test for paired samples was used to test the difference in endocrine parameters among time points. In addition, OT and AVP concentrations in the two groups of dogs were compared using Mann–Whitney *U* tests. The OT and AVP concentrations and the duration and relative frequency of behaviors were presented as median. *p*-values ≤ 0.05 were deemed statistically significant. Statistical analyses were performed with IBM SPSS Statistics 25.0.

## 3. Results

### 3.1. Behavioral Responses

**Figure 2:**
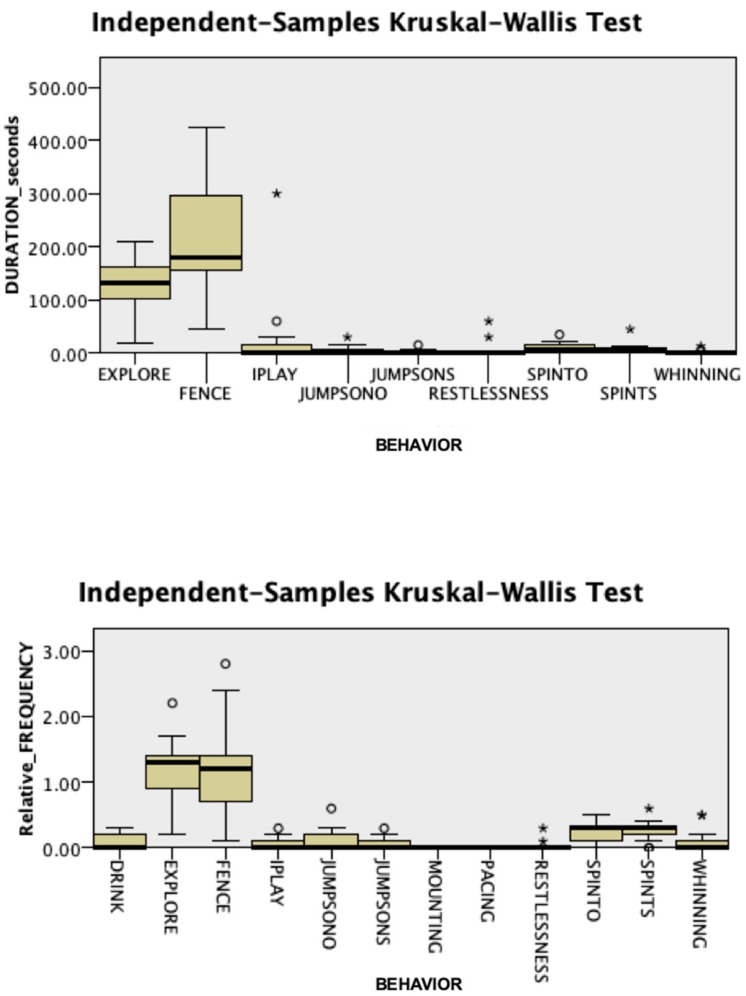
Duration and relative frequency, expressed as the number of occurrences per minute, of the behaviors observed during the acclimatization period in the Case group (N = 13). FENCE: Attention oriented to the fence; IPLAY: Individual play; JUMPSONO: Jumps on owner; JUMPSONS: Jumps on the stranger; SPINTO: Spontaneous interactions with the owner; SPINTS: Spontaneous interactions with a stranger.

**Figure 3:**
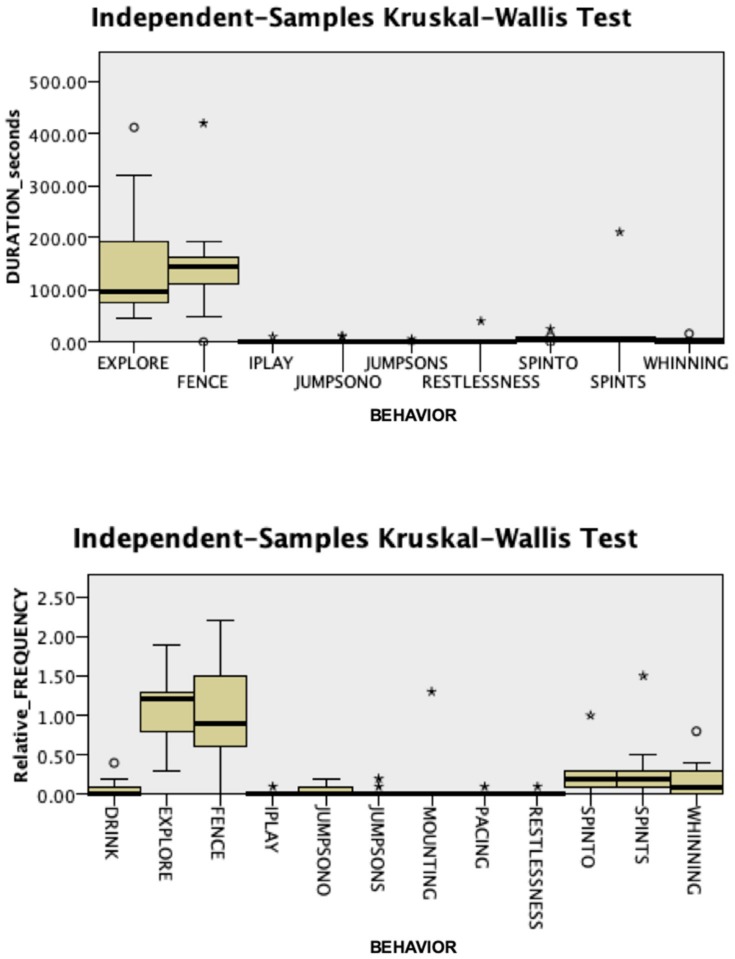
Duration and relative frequency, expressed as the number of occurrences per minute, of the behaviors observed during the acclimatization period in the Control group (N = 13). FENCE: Attention oriented to the fence; IPLAY: Individual play; JUMPSONO: Jumps on owner; JUMPSONS: Jumps on the stranger; SPINTO: Spontaneous interactions with the owner; SPINTS: Spontaneous interactions with a stranger.

Intra-observer and inter-observer reliability were confirmed, with a Cronbach’s α of 0.995 and 0.997, respectively. No significant differences were found in behavioral responses during the acclimatization phase between groups (Mann–Whitney *U* test, *p* > 0.05). As shown in Figure 2 and Figure 3, in this phase, attention directed towards the fence and exploring were the most frequent behaviors and had the greatest total duration in both Case (duration: Kruskal–Wallis test, χ^2^ = 52.803, *p* = 0.001; relative frequency: Kruskal–Wallis test, χ^2^ = 93.552, *p* = 0.001) and Control dogs (duration: Kruskal–Wallis test, χ^2^ = 37.895, *p* = 0.001; relative frequency: Kruskal–Wallis test, χ^2^ = 97.349, *p* = 0.001). Behaviors oriented towards the fence and standing by the fence were not sufficiently expressed to be analyzed in this phase.

**Figure 4:**
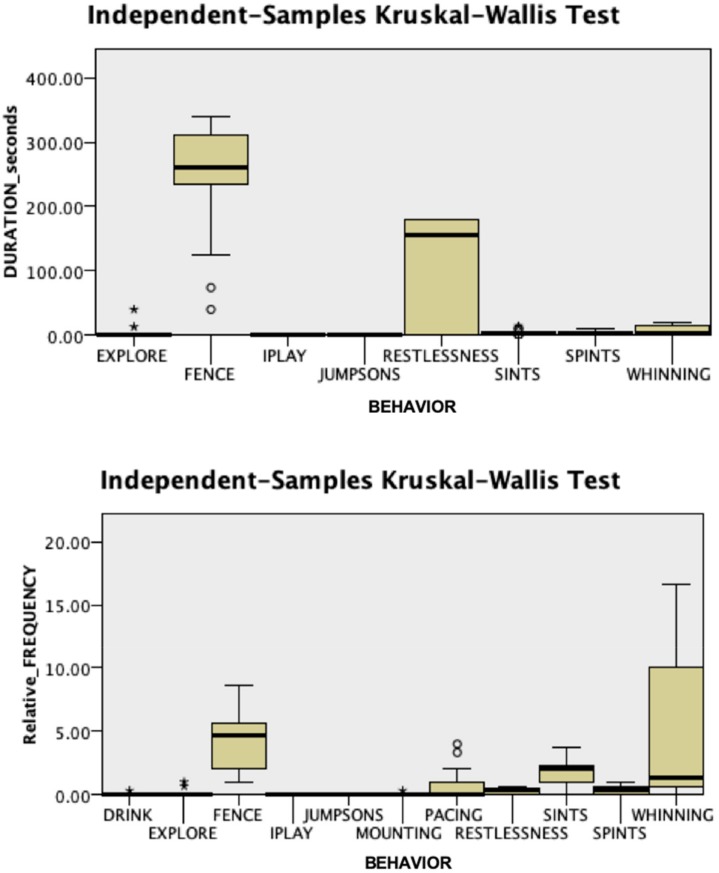
Duration and relative frequency, expressed as the number of occurrences per minute, of the behaviors observed during the separation period in the Case group (N = 13). Fence: Attention oriented to the fence; IPLAY: Individual play; JUMPSONO: Jumps on owner; JUMPSONS: Jumps on the stranger; SPINTO: Spontaneous interactions with the owner; SPINTS: Spontaneous interactions with a stranger.

**Figure 5:**
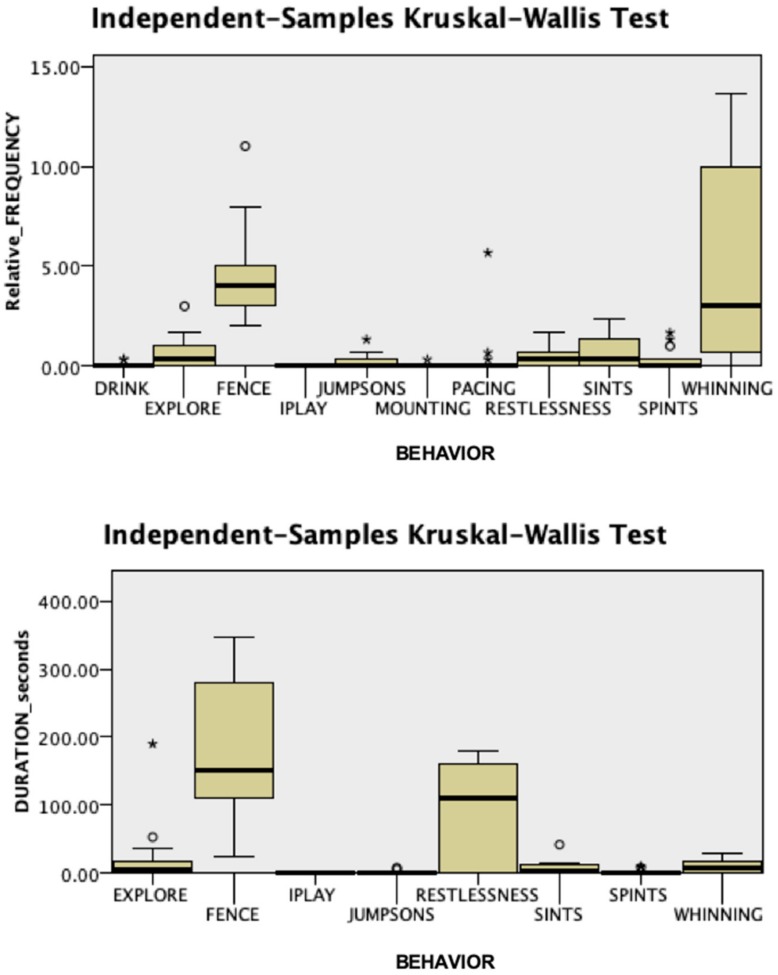
Duration and relative frequency, expressed as the number of occurrences per minute, of the behaviors observed during the separation period in the Control group (N = 13). FENCE: Attention oriented to the fence; IPLAY: Individual play; JUMPSONO: Jumps on owner; JUMPSONS: Jumps on the stranger; SPINTO: Spontaneous interactions with the owner; SPINTS: Spontaneous interactions with a stranger.

The groups differed significantly during the separation phase (Figure 4 and Figure 5). Dogs in the Case group showed significantly less exploration (duration: 0 vs. 6, Case vs. Control; Mann–Whitney U = 122.500, *p* = 0.05; relative frequency: 0 vs. 0.33, Mann–Whitney U = 125.000, *p* = 0.039) and spent significantly more time standing by the fence (duration: 260 vs. 151, Case vs. Control; Mann–Whitney U = 36.000, *p* = 0.012, Figure S1) than Control dogs. In addition, at T1, we recorded significantly more frequent attempts by the veterinary behaviorist to interact with Case dogs (relative frequency: 2 vs. 0.33, Case vs. Control; Mann–Whitney U = 40.000, *p* = 0.022) than with controls. During dog-owner reunion, Case dogs were significantly more persistent than dogs from the Control group in jumping up on the stranger (duration: 10 vs. 0, Case vs. Control; Mann–Whitney U = 185.000, *p* = 0.004; Supplementary Video S3), with the veterinary behaviorist as the only target of this behavior.

### 3.2. Endocrine Responses

**Figure 6:**
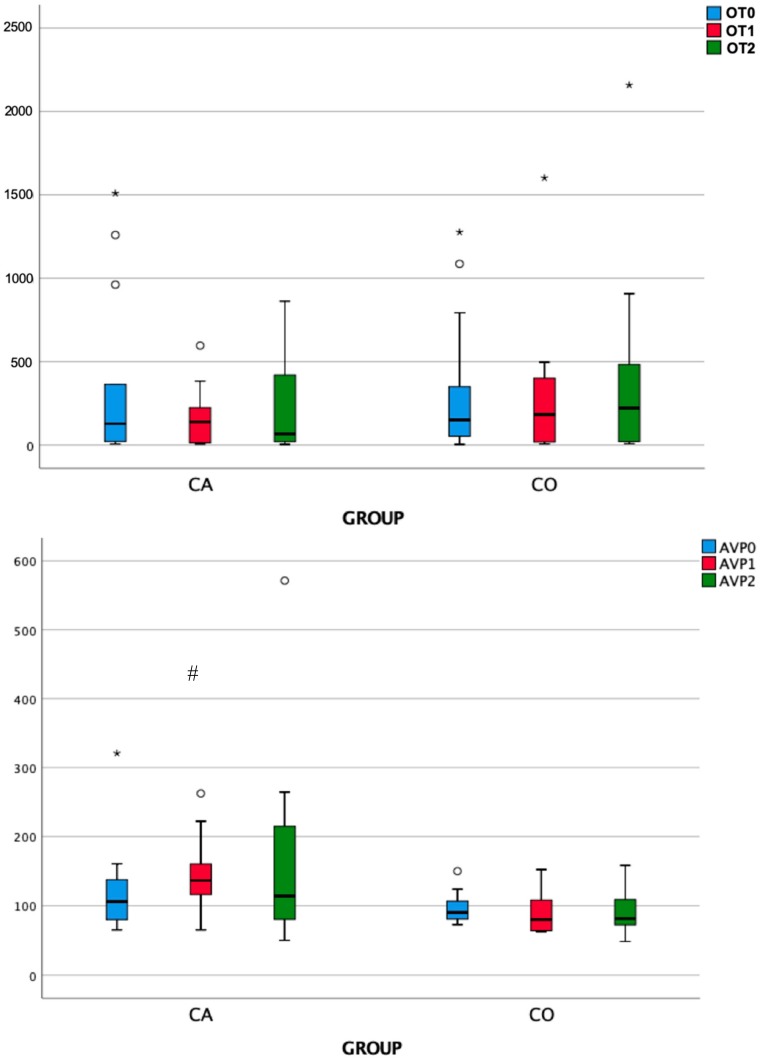
Concentrations of oxytocin (OT) and vasopressin (AVP) measured in saliva before (T0), immediately (T1), and 10 min after (T2) separation from the owner. CA: Case group, N = 13. CO: Control group, N = 13. Salivary AVP concentrations showed a statistically significant difference between groups at T1 (^#^, *p* < 0.05).

As shown in Table 2 and Figure 6, the analysis of salivary OT and AVP revealed non-significant differences among time points within each group. However, at T1, AVP concentrations were significantly higher in the Case group than in the Control group. In addition, there was a trend, although not statistically significant, towards lower OT concentrations at T1 and T2 and higher AVP concentrations at T2 in Case dogs than in controls.

## 4. Discussion

This study aimed to determine whether there were differences in specific behaviors and endocrine responses between dogs affected by separation distress at home and behaviorally normal dogs while experiencing a potentially anxiogenic situation (separation from the owner in a novel environment and the presence of two strangers). We found that during the initial acclimatization phase, when the owner was still present, dogs with SRP behaved similarly to normal dogs. They were mainly explorative, as reflected by the higher frequency and longer duration of time spent in investigating the physical environment investigation time than in the other behaviors. Thus, dogs in both groups exhibited a similar attachment style, showing that they viewed the attachment figure (the owner) as a secure base for exploration of the novel environment [2]. This finding did not conform to the traditional vision, which considers dogs with separation distress as having excessive attachment towards their owners [40,41]. However, it agreed with what was more recently reported by Parthasarathy and Crowell–Davis [4], namely, that separation distress is not correlated with hyper attachment, confirming that these dogs were confident with the novel environment and strangers. Regarding the behavioral responses observed during the separation episodes, contrary to what was expected based on the results by Parthasarathy and Crowell–Davis [4], the patterns of behavior were different for the two groups in our study. Dogs in the Case group were significantly less explorative and more persistent in passive behaviors aimed at maintaining proximity to the owners, such as staying near the fence by the exit, than controls. According to Topál et al. [37], who analyzed the attachment relationship of behaviorally typical dogs with their owners, the fact that, during the separation phase, this behavior was not reduced by the presence of a stranger, despite her attempts to relate positively, might suggest that dogs with SRP have an especially strong preference for their owners in stress situations. This searching response has been observed in children [42] and non-human primates [43,44] and is regarded as aimed at maintaining the comforting bond of attachment. Nevertheless, dogs in the study by Topál et al. [45] showed a tendency to seek and maintain contact with the returning owner but not the stranger. Conversely, in our study, dogs with separation distress tended to show greater persistence in investigating the veterinary behaviorist (as suggested by more time spent jumping up) during post-separation reunion than did Control (CO) dogs, and this could also be seen as indirect evidence that these dogs experienced a higher level of anxiety during the separation. In fact, although we could not exclude the possibility that this was a side effect of dogs with separation distress needing to spend more time in proximity to the owner, who was sitting near the stranger, another possible explanation could be put forward. The veterinary behaviorist was the stranger who had made attempts to relate to dogs during the separation phase when proximity to their attachment figure was no longer possible. Those efforts might have made her a target of exploration for the SRP dogs in the reunion phase when contact with the attachment figure was re-established. This would also explain why the dogs’ jumping up behavior was directed only towards the veterinary behaviorist and not the camerawoman. Indeed, even if our experimental design was supposed to lead to calmer dogs compared to that used by Topal et al. [45] (e.g. a 10 vs. 4 minutes acclimatization period; dog vs. stranger initiated contact in the separation phase), dogs in the study by Topal et al. [45] were all behaviorally normal, and therefore less prone to anxiety than our Case dogs. More research is needed to understand whether behavioral reactions of a dog while alone (such as reduced exploration and increased seeking of proximity to the owner), or even after the owner’s return, might be used as effective indicators of the presence of separation distress disorders.

Notably, salivary AVP concentrations were significantly higher in Case dogs than Control dogs immediately after the end of the separation period (T1). The timing with which OT and AVP reach saliva is not well understood, but they appear faster than other salivary hormones (e.g., cortisol), which reach peak concentrations in saliva ∼10 min after those in blood [31]. Previous studies have shown effects at a minimum time delay of 10 min [13,31]. However, other studies revealed early increases in salivary OT concentrations in nursing mothers and dams [31,46]. In the study by MacLean et al. [31], in particular, a large and statistically significant increase in salivary OT was detected in dams from baseline, at the end of a pre-test separation from their litters, to nursing 3 min later. This effect has been interpreted as reflecting an anticipatory rise in salivary OT. Similarly, it is possible that, in our study, the significant between-group difference observed in salivary AVP at 3 min partially reflected a rapid anticipatory response in SRP dogs. Given that non-social fears, such as fear of novel situations/environments, are common comorbidities of separation anxiety in dogs [17,47], we chose only dogs that did not present signs of stranger-directed aggression or fear of strangers or novel environments. Therefore, jointly with the fact that all dogs acted normal during the acclimatization period, the difference at 3 min was unlikely to be the result of stress at the start of the experiment in a novel environment. It is worth noting that, at T2, Case dogs still had higher concentrations of AVP than Control dogs, although the difference was not statistically significant due to the relatively high standard error of the mean. Although we could not be certain, it is possible that separation from the owner accounted also for this difference in salivary concentrations. 

This was an intriguing outcome because central AVP, particularly that released within the amygdala, has been shown to be involved in the generation of passive coping strategies for acute stress in rodent models [37,48] through processes that would be mediated by the V1a and V1b receptors [48].

Both OT and AVP are synthesized in the hypothalamus, primarily in large magnocellular neurons situated in the supraoptic and paraventricular nuclei, and secreted from their axons, which are projected to the neurohypophysis, into the general circulation (for example, during labor or imbalance of water homeostasis) [48]. Vasopressin molecules that have been released in this way are, for the most part, prevented from re-entering the central nervous system via the blood-brain barrier [49]. In parallel, AVP and OT are also secreted within the brain, from the dendrites of the same neurons, in a manner regulated semi-independently of axonal release [50]. Following secretion, these peptides diffuse throughout the extracellular space, serving as neuromodulators for surrounding brain tissue [51]. As above, dendritic secretion of AVP has been shown to be of central importance in animal models of anxiety disorders [48], while axonal secretion has been shown to affect fear responses in mice [52], probably by regulating stress responses through the hypothalamic–pituitary–adrenal axis [53].

In our study, we could detect only an increased peripheral AVP level immediately after separation-induced social stress in SRP dogs compared to normal dogs, but central concentrations of AVP were likely increased as well. In fact, plasma and salivary AVP, measured using ELISA kits, were found to be moderately correlated in humans in one study [54], and plasma AVP concentrations significantly and positively predicted cerebrospinal fluid AVP concentrations in human neonates in another study [55]. Thus, these two studies provided preliminary support for the use of salivary AVP ELISA measurement as a proxy for brain AVP activity, at least, in humans. Future studies are now required to determine the relationship between behavioral measures and AVP concentrations in both the central and peripheral compartments in dogs.

It is worth mentioning that interest in using V1b antagonism to treat anxiety disorders has been investigated. Clinical trials in humans failed, with the V1b receptor antagonist SSR149415 not being useful for the treatment of generalized anxiety disorder [56]. However, two newly synthesized V1b receptor antagonists—TASP0233278 and TASP0390325—have shown potential benefits in rodent models [57]. Future studies could examine whether this therapeutic approach might also benefit dogs diagnosed with SRP. The antidepressants—clomipramine and fluoxetine—which act primarily as serotonin reuptake inhibitors, are currently approved for the treatment of canine separation distress [58]. As more evidence is collected on the role of AVP in the pathophysiology of SRP, future drugs targeting the vasopressinergic system would offer treatment options for canine separation distress therapy.

Finally, although AVP is often anxiogenic, the closely related nonapeptide OT often has anxiolytic effects [59] and may reduce the stress of negative social interactions [60]. The concentrations of OT that we reported here with a sample size of 26 dogs (13 behaviorally normal, 13 with SRP) did not reach statistical significance over time, although a trend towards a reduction (and an increase in AVP concentrations) was observed during and even after the separation from the owner in dogs from the Case group. This could also be a type II error-related false-negative result due to the small sample size. Future research, exploring in a larger sample size whether both OT and AVP respond dynamically to this potentially anxiogenic situation, might help detect a significant effect.

## 5. Conclusions

In conclusion, this study showed that, when placed in a novel environment, dogs presented different behavioral and endocrine responses to a short separation from the owner, followed by the reunion, depending on whether they suffered from separation distress at home. Dogs with SRP became more distressed than CO dogs when the owner was gone for a short time and left them in the company of two unfamiliar persons. They were less able to mediate their reactions in such a stressful situation, showing more passive coping strategies aimed at seeking proximity to the owner while he/she was absent, and had significantly higher salivary concentrations of AVP at the very end of this phase, which are two responses that have been previously associated in other animal models of social separation. Although these results are preliminary and should, therefore, be interpreted with caution, the differences observed between groups in both behavioral and endocrine responses during and after the separation lead the way to further exploration of the use of salivary AVP as an early, non-invasive biomarker of canine anxiety-related disorders and support AVP antagonism as a potential new mechanism-based therapeutic approach. According to Thielke and Udell [61], improved methods of treating SRP in dogs would not only benefit human-dog relationships but also potentially contribute to the decreased surrender of dogs to shelters by reducing the effort needed to modify this behavior problem successfully.

## Figures and Tables

**Table 1:** List of behaviors and definitions used in the study. F = frequency (number of occurrences); D = duration (s).

Behaviors	Description	Measured Values (F/D)
**Social behaviors**		
Jumping up	Both of the dog’s forelegs were out of contact with the ground, regardless of the position of the hind legs; the dog was in proximity to a person. The dog might also be entirely on a person’s lap	F, D
Spontaneous interactions	Staying close to and seeking attention and physical contact (nuzzling or pawing for attention, soliciting petting) from the owner or the stranger	F, D
Mounting	Sexual mounting of people or inanimate objects	F, D
**Non–social behaviors**		
Explore	Activity directed towards physical aspects of the environment, including sniffing, visual inspection, and gentle licking	F, D
Individual play	Any behavior performed vigorously or at a galloping gait and directed towards an object when clearly not interacting with any human; these play behaviors included chewing, biting, shaking from side to side, scratching or batting with the paw, chasing rolling balls, and tossing objects using the mouth	F, D
Standing by the fence	Standing close to the fence (<1 m), regardless of whether the face was oriented towards the exit	F, D
Attention oriented towards the fence	Staring fixedly at the fence, either when close to it or from a distance	F, D
Behaviors oriented towards the fence	All activities, resulting in physical contact with the fence, including scratching the gate with the paws, jumping on the fence, and pulling on the fence with the forelegs or mouth.	F, D
Restlessness	A feeling of agitation expressed by continual motion; changing the state of locomotion; digging (scratching the floor with the forepaws in a way that is similar to when dogs are digging holes)	F
Drinking	Taking in fluids by lapping up water from the bowl with the tongue	F
Whining	High-pitched vocalization	F
Pacing	Increased motor activity, walking or running around without exploring the environment	F

**Table 2:** Salivary concentrations of oxytocin (OT) and vasopressin (AVP).

**Group**	**OT0**	**OT1**	**OT2**	**Friedman test**	
				χ2	P
**Case**	127.87	138.79	67.04	3.231	0.199
**Control**	149.99	183	221.60	0.462	0.794
**Mann–Whitney U test**	86	98	100		
**Mann–Whitney U test p**	0.960	0.511	0.448		
**Group**	**AVP0**	**AVP1**	**AVP2**	**Friedman test**	
				χ2	P
**Case**	105.97	136.61	114	2.923	0.232
**Control**	90.40	80.12	81.53	3.231	0.199
**Mann–Whitney U test**	71	29	55		
**Mann–Whitney U test p**	0.511	0.003	0.139		

## Supplementary Materials

The following are available online at https://www.mdpi.com/2076-2615/9/12/1033/s1, **Figure S1:** A photo of a dog from the Case group who is standing by the fence during the 3-minute separation phase (T1). Photo by Valentina Sammartano. **Table S1:** Main characteristics of the dogs involved in the study. **Video S1:** A dog is showing signs of stress while the owner is out of the arena and the veterinary behaviorist is engaging the dog in friendly interaction, including gently petting her and speaking to her in a calm tone. **Video S2:** A dog is exhibiting signs of severe distress and anxiety; consequently, the owner is asked to come back, and the test is stopped. **Video S3.** A dog in the Case group persists in jumping up on the veterinary behaviorist during the post-separation reunion phase.
