# Supplementary material for: Salivary Vasopressin as a Potential Non–Invasive Biomarker of Anxiety in Dogs Diagnosed with Separation–Related Problems
Source: Animals (Basel). 2019 Nov 26;9(12):1033. doi: 10.3390/ani9121033 (PMC6941168; doi:10.3390/ani9121033)

Table S1 Main characteristics of the dogs involved in the study.

Dog_ID	Owner_gender	Dog_Age (years)	Breed	Dog_Gender	Sexual_status	Dog_source	Separation_distress
1	male	2,0	mixed	male	intact	shelter	YES
2	female	4,0	pure	female	desexed	breeder	YES
3	female	5,0	pure	female	intact	shelter	YES
4	female	2,0	pure	female	intact	breeder	YES
5	female	3,0	mixed	male	desexed	found	YES
6	male	2,0	mixed	female	desexed	found	YES
7	female	2,0	mixed	male	intact	found	YES
8	male	2,5	mixed	female	desexed	friend/relative	YES
9	male	2,0	mixed	female	intact	found	YES
10	female	4,0	pure	male	intact	breeder	YES
11	male	1,5	pure	male	intact	breeder	YES
12	female	2,0	mixed	male	desexed	found	YES
13	female	1,5	mixed	female	intact	shelter	YES
14	male	5,0	pure	male	desexed	friend/relative	NO
15	male	1,0	pure	female	intact	breeder	NO
16	male	6,0	mixed	male	desexed	friend/relative	NO
17	female	8,0	mixed	female	desexed	found	NO
18	female	4,0	mixed	male	intact	found	NO
19	female	5,0	pure	female	intact	breeder	NO
20	male	2,0	mixed	male	intact	found	NO
21	female	5,0	pure	male	desexed	shelter	NO
22	female	6,0	mixed	male	desexed	friend/relative	NO
23	female	8,0	mixed	male	desexed	found	NO
24	female	8,0	mixed	female	desexed	found	NO
25	male	2,0	pure	male	intact	breeder	NO
26	female	2,0	mixed	female	intact	found	NO

Figure S1. A photo of a dog from the Case group who is standing by the fence during the 3-min separation phase (T1). Photo by Valentina Sammartano.

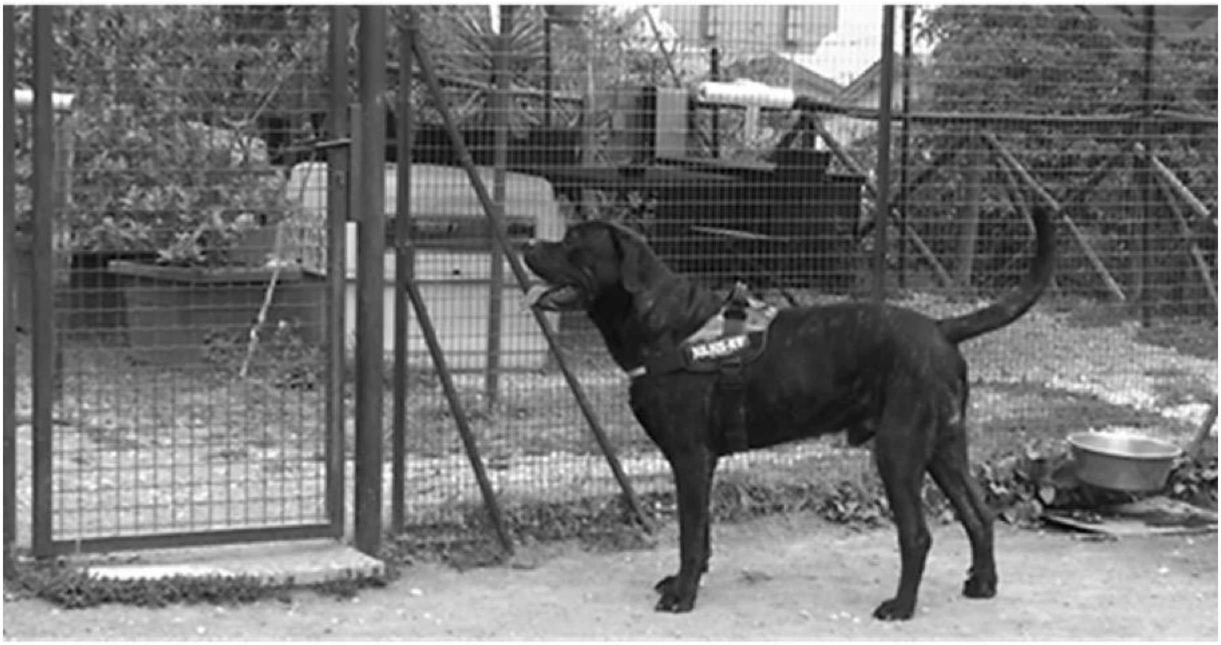

Supplement: Supplementary file 1 [file animals-09-01033-s001.pdf]
